# Strategies for the Construction of Mouse Models With Humanized Immune System and Evaluation of Tumor Immune Checkpoint Inhibitor Therapy

**DOI:** 10.3389/fonc.2021.673199

**Published:** 2021-04-29

**Authors:** Wenwen Guo, Caiqin Zhang, Tianyun Qiao, Jumei Zhao, Changhong Shi

**Affiliations:** ^1^ Division of Cancer Biology, Laboratory Animal Center, Fourth Military Medical University, Xi’an, China; ^2^ School of Basic Medical Sciences, Medical College of Yan’an University, Yanan, China; ^3^ Department of Thoracic Surgery, Tangdu Hospital, Fourth Military Medical University, Xi’an, China

**Keywords:** humanized immune system, mouse model, tumor, immunotherapy, combination therapy

## Abstract

Immunotherapy has been used as a first-line treatment for a variety of advanced tumors, allowing remarkable progress to be made in cancer treatment. Nonetheless, only a small number of patients can benefit from immune checkpoint inhibitor monotherapy. To improve the effect of immunotherapy, the underlying mechanism of combination therapy was investigated in the context of an intact human tumor immune microenvironment using mice with a human immune system (HIS) bearing human tumors. Herein, we summarize and discuss strategies for the development and use of HIS mice models in tumor immunotherapies. Most importantly, this review proposes a method of t11umor identification and classification in HIS mice based on the tumor-infiltrating lymphocytes and PD-L1 expression, and according to this classification, we propose different combination treatment strategies that can be utilized to enhance the effect of immunotherapy. Thus, we provide effective experimental schemes for tumor immunotherapy in HIS mice models.

## Introduction

Recently, following surgery, radiotherapy, chemotherapy, and molecular targeted therapy, immunotherapies have emerged as one of the most promising approaches to cancer treatment (1). The first immune checkpoint inhibitor (ICI), ipilimumab, was approved by the US Food and Drug Administration in 2011 as a first-line treatment for melanoma; since then, ICI has been used for the clinical treatment of various cancer types, including non-small cell lung cancer (NSCLC), renal cell carcinoma, head and neck squamous cell carcinoma, and Hodgkin’s lymphoma, thus displaying good prospects for clinical application (2, 3). However, only a small number of patients are sensitive to immunotherapy, mainly owing to their high levels of immune checkpoint protein expression and extensive immune cell infiltration (4). Therefore, the development of methods to improve the effect of immunotherapy in non-sensitive patients became the focus of immunotherapy research.

To expand the benefits of the immunotherapy recipients, research on novel immunotherapy or immunotherapy-based combination therapy is crucial (5). The need for more effective combination therapies to expand the patient population benefiting from immuno-oncology agents and overcoming treatment resistance has been increasingly recognized; however, significant work is required to develop a comprehensive understanding of the immune microenvironment of different tumors and subtypes prior to designing reasonable combination treatment strategies (6). Conversely, numerous combined treatments require sufficient preclinical evidence before commencing clinical trials, thus emphasizing the importance of building a solid preclinical drug evaluation platform (7).

Mice exhibiting a human immune system (HIS) and bearing human tumors could provide an ideal preclinical model for cancer immunotherapy research (8). However, because numerous factors influence the successful construction of suitable HIS mouse models, it is important to select the most suitable method for their construction. In this review, we comprehensively discuss how to choose the appropriate construction method, mouse strain, donor, and tumor transplantation plan to successfully establish an ideal preclinical model for follow-up immunotherapy research. Furthermore, we discuss the role of the tumor immune microenvironment and evaluate the effect of tumor immunotherapy by determining the expression level of immune checkpoint proteins and the degree of immune cell infiltration. Based on these considerations, we suggest different combined strategies to enhance the effect of immunotherapy and provide effective experimental schemes for tumor immunotherapy with humanized mouse models.

## Subsections Relevant for the Subject

### Establishment of HIS Mouse Model

Humanized mice that can recapitulate a partially functional HIS represent the best preclinical model for immunotherapy, allowing for optimum characterization of the tumor immune microenvironment and predicting patient responses to immunotherapy. However, there are numerous factors influencing the construction of such model.

### Model Construction Methods

**Figure 1:**
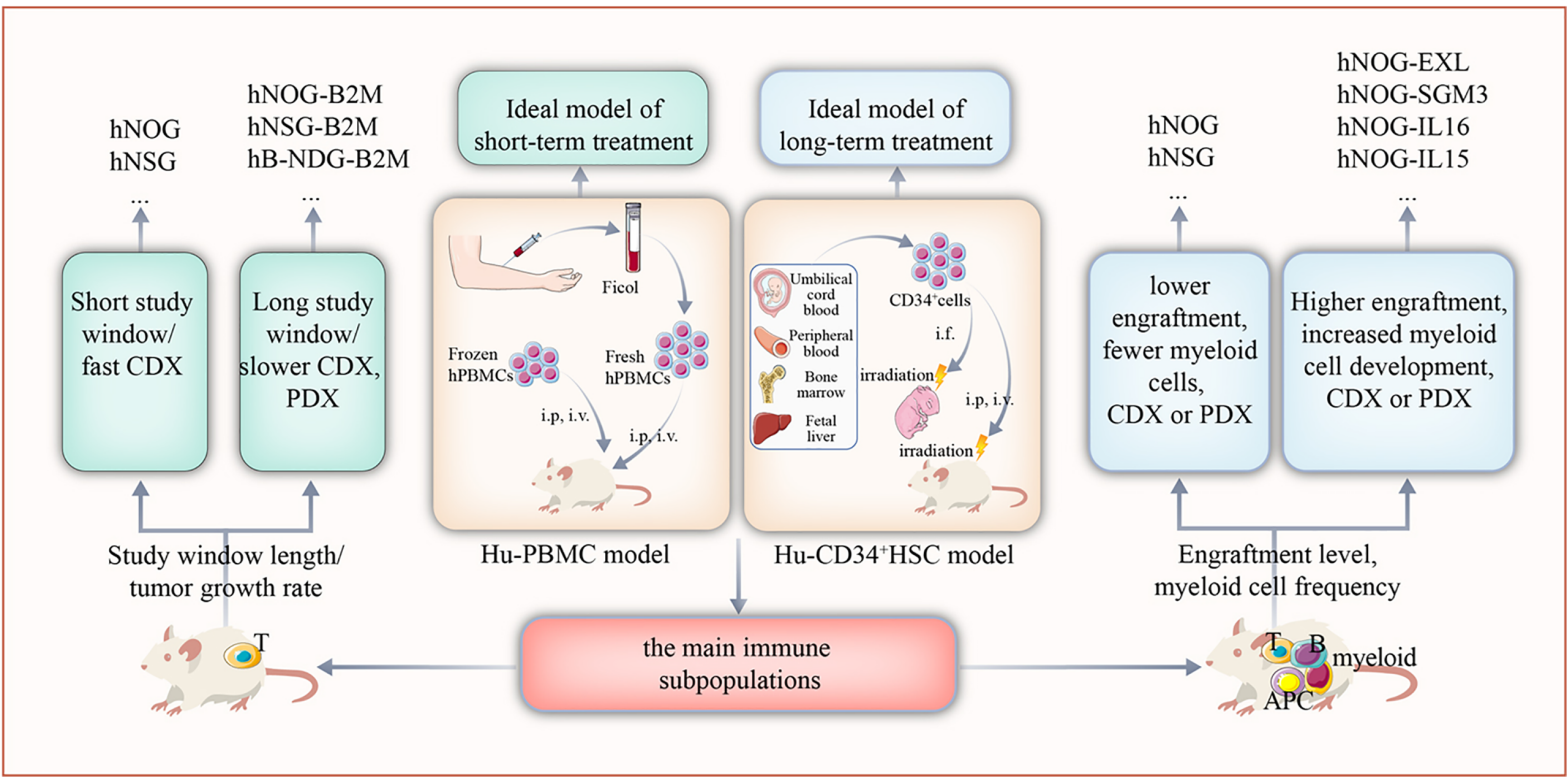
The selection of appropriate humanized mouse model according to the design of the study. Based on the target immune cell subsets of the study, length of the study window, and growth rate of the tumor, the Hu-PBMC or Hu-CD34^+^ HSC model should be used.

According to the differences in construction methods, HIS mouse models are mainly divided into the human peripheral blood mononuclear cell (Hu-PBMC) model and the CD34^+^ hematopoietic stem cell (Hu-CD34^+^ HSC) model (9). The construction time, treatment window, and immune cell subsets of the two models are quite different; thus, it is of great importance to choose the appropriate preclinical model according to the experimental design (**Figure 1**).

The construction method of the Hu-PBMC model is simple and fast. By transplanting PBMCs from healthy adults into severe combined immunodeficient mice, the functions of some human T cells are successfully reconstructed in approximately 2 weeks (10); however, this model was shown to lack other immune cell subsets, such as B cells and natural killer (NK) cells (11). Furthermore, weight loss and hair depilation were shown to occur 3–5 weeks following transplantation, as a result of the graft-versus-host reaction (GvHD). Therefore, the experimental window of the model is only approximately 4–6 weeks (10). Overall, the Hu-PBMC model represents an ideal preclinical model for short-term immunotherapy studies.

The Hu-CD34^+^ HSC model is established by transplanting human CD34^+^ hematopoietic stem cells from umbilical cord blood into irradiated immunodeficient mice. The construction time of this model extends to 8–12 weeks. HSCs differentiate and develop into whole line immune cell subsets, including T, B, and NK cells, and a long-term efficacy test can be carried out for up to 45 weeks (12). However, the development of myeloid cells in this model is limited. Thus, the Hu-CD34^+^ HSC model represents an ideal preclinical model for long-term immunotherapy studies.

### Mouse Strains

The following mouse strains are currently widely used to generate HIS mouse models: NOD/shi-SCID γ c-/- (NSG)-(NOG), NOD/SCID γ c-/- (NSG) (13), BALB/c-Rag2-/-IL2rg-/- (BRG) (14), and NOD-Prkdcem26Cd52Il2rgem26Cd22/Nju (NCG) (15). It is recognized as the mouse with the highest degree of immune deficiency and is thus considered the most suitable for human PBMC and human CD34^+^ HSC transplantation.

As the GvHD is rapidly induced in an Hu-PBMC model, beta-2 microglobulin (B2m) knockout mice have been developed to solve the problem of the short research window caused by this model. In addition, to address the limited development of myeloid cells in Hu-CD34^+^ HSC models, mice model expressing human IL-3, GM-CSF, or SCF, such as the NSG-SGM3, NOG-EXL, or NOG-IL-6, have been established (16). These mice can support the differentiation and development of myeloid cells, including granulocytes, monocytes, and macrophages. Moreover, B-NDG hTHPO (B-NDG-Thpotm1 (THPO)/Bcgen) mice were found to express human THPO protein, which increased the level of implantation of human bone marrow in the host and improved the multidirectional differentiation of hematopoietic cells (17).

### Cell Donors

Differences have been reported in the reconstruction effect of PBMC/CD34^+^ HSCs from different donor sources. These differences are indicated by the different levels and reconstruction pace of PBMC/CD34^+^ HSCs from different donors in the same mouse strain. Furthermore, it was reported that humanized mouse models constructed using PBMCs or CD34^+^ HSCs from distinct donors have different responses to ICI, and that this responses are consistent with the clinical results (18). To minimize the influence of donor differences on the experimental data, humanized mice from at least two donors are usually randomly distributed in each group.

The construction of a humanized mouse model requires transplantation of an optimal number of immune cells. In general, the number of transplanted human CD34^+^ HSCs is approximately 1 × 10^4^–2 × 10^5^, while in most experimental studies 1 × 10^5^ transplanted cells are used (19). Considering that sourcing CD34^+^ HSCs is difficult and that the number of cells obtained is relatively small, expanding the number of cell *in vitro* can lead to improved outcomes. Although the number of CD34^+^ HSCs can be increased by 10–20 fold *in vitro*, this leads to a loss of their characteristic stemness (20). For constructing an Hu-PBMC model, the optimum number of cells is 1 × 10^7^ PBMCs. In short, implantation of an excessive number of CD34^+^ HSCs may lead to anemia, while implantation of an excessive number of PBMCs will accelerate the GvHD (21). Alternatively, an insufficient number of transplanted cells may lead to a low transplantation rate or transplant failure.

Two types of HIS mouse models can be reconstructed using PBMC/CD34^+^ HSCs following cryopreservation and resuscitation. Some studies have confirmed that fresh and cryopreserved cells have no effect on the success rate of immune reconstruction (12), thus allowing prescreened donors to be used for immune resuscitation in later experiments, which is crucial for long-term research.

### Tumor Implantation Strategy

To further benefit from the research window of HIS models, the time of tumor and donor transplantation must be optimized so that the tumor can reach the treatment volume while the model is successfully reconstructed. Hu-PBMC mice models might develop the GvHD before treatment initiation if the tumor is transplanted too late; therefore, tumor cell lines should preferably be inoculated before PBMC transplantation. Because the tumor growth rate of patient-derived xenograft (PDX) model is slow and it might take several months until PDX can be treated (22), the optimal method is to first establish the PDX model and then carry out the reconstructive process using PBMCs. In summary, the optimal inoculation scheme needs to be adjusted according to the tumor growth kinetics to maximize the window of the study.

In addition, previous studies suggest that human leukocyte antigen (HLA) matching might be important for the successful establishment of tumor xenografts (23). In an Hu-CD34^+^ HSC model, while partial HLA-matched PDX tumors were implanted, the tumor growth was not significantly different compared with that of non-HLA-matched NSG mice (24). In another study, approximately 60–80% of PDX-implanted HIS mice developed tumors, regardless of whether the donor was HLA-matched or not (25). Overall, both studies converge on the lack of effect of the matching status of HLA on the response rate of ICI.

## Tumor Identification and Classification in HIS Mice

After construction of a suitable HIS mouse bearing a humanized tumor, the tumor needs to be identified and classified to predict the immunotherapeutic effects and devise a targeted treatment plan. Clinically, patients with different tumors and subtypes have different response rates to immunotherapy. Because traditional TNM (Tumor, Lymph node, Metastasis) staging cannot explain this phenomenon, it is important to find new methods to identify and classify which tumors are sensitive to immunotherapy (26). Numerous preclinical and clinical studies indicate that the effect of immunotherapy is related to the expression level of immune checkpoint molecules in the tumor microenvironment (TME) (6, 27).

### Expression of Immune Checkpoint Molecules in Tumors

Immune checkpoint molecules are the main participants in maintaining individual immune homeostasis by regulating the level and duration of physiological immune responses. Different immune checkpoints are essential for limiting tissue damage and promoting self-tolerance by inhibiting the inflammatory activity of T cells. The most common immune checkpoint molecules are PD-1, LAG-3, TIM-3, and CTLA-4 (28). Tumor cells can produce inhibitory ligands that can bind to immune checkpoint molecules, thus limiting the normal antitumor immune response and assisting immune escape. Therefore, high immune checkpoint molecule expression is undoubtedly associated with poor prognosis. Clinically, by targeting these immune checkpoint molecules, especially the PD-L1/PD-1 and CTLA-4 pathways, exhaustive immune cell death can be blocked. This will allow the strength of the host immune system to enhance endogenous antitumor activity and achieve a good therapeutic effect by indirectly killing tumor cells. The PD-L1 expression level is an important biomarker for predicting the effect of immunotherapy and identifying potential immunotherapy beneficiaries (29). In a phase III keynote-024 trial, patients with advanced NSCLC and high PD-L1 expression (Tumor Proportion Score, TPS ≥ 50%) who were treated with pembrolizumab monotherapy had longer progression-free survival than patients treated with platinum-based chemotherapy (30). Other studies reported that high PD-L1 expression levels were correlated with a high effective rate and median survival time (31). In view of these results, it is necessary to analyze the expression level of PD-L1 and other immune checkpoint molecules in the Hu-CDX and PDX models to predict the effects of treatment and determine a follow-up treatment plan.

### Immune Cell Infiltration in Tumors

Tumor immunotherapy has demonstrated that immune cells, especially T cells, are the main cells responsible for eliminating tumor cells. However, only a small number of cancer patients benefit from the TME treatment strategy. As a major component of the TME, the level of ICI also contributes to tumor response to immunotherapy. Therefore, a better understanding of the role of innate and adaptive immune cells in the TME is essential for deciphering immunotherapy mechanisms, defining predictive biomarkers, and identifying new therapeutic targets.

Previously, tumor immunotherapy has mainly focused on how to promote the killing of tumor cells by enhancing the immune response; however, the targeted effect of this approach is limited, and patients might experience side effects such as the development of autoimmune diseases. Recent evidence demonstrates that tumor patients have antitumor immune T cells and are in a state of immunosuppression owing to a variety of tumor immune escape mechanisms (32, 33). Chen & Sanmamed suggested that restoring the function of immune cells in the tumor immune microenvironment is more important than enhancing the tumor immune response (32). The successful application of immunotherapy methods, such as of ICI, supports this view. Thus, it is critical to analyze and normalize the level of immune cell infiltration in the tumor immune microenvironment. Accordingly, Galon et al. proposed a quantitative immune scoring (IS) method for two types of lymphocytes (CD3 and CD8) in the tumor center and an invasive margin to evaluate the immune cell infiltration level in the TME (33). A value of 1 represented a high density of positive cells, while 0 represented a low density. The total immune scores of CD3^+^ and CD8^+^ cells in the tumor center and margins were added to obtain the total immune score (0–4), which was positively correlated with survival time. The predictive value of IS for disease-free survival (DFS), tumor-specific survival rate, and overall survival (OS) time was better than that of the gold standard TNM staging system.

According to the levels of immune cells, a tumor classification method based on immunity rather than TNM staging has been proposed for the first time. Conversely, the terms of “hot” or “cold” have increasingly been used to classify tumors according to their degree of immune cell infiltration (high and low immune scores, respectively) (33). By stratifying the tumor based on T cell infiltration, the immune score might represent a powerful method to identify the level of immune cell infiltration in the TME.

### Tumor Immune Microenvironment Classification

Not all patients with positive PD-L1 expression respond well to ICI, suggesting that other microenvironmental factors might play an important role in the immunotherapy response (34). As previously mentioned, the degree of TME infiltration by tumor-infiltrating lymphocytes (TILs) is also associated with the clinical efficacy of anti-PD-1/PD-L1 therapy. Furthermore, previous studies have discovered that the better efficacy of ICI is associated with a higher tumor mutation load, more abundant new antigens, and higher microsatellite levels (35, 36).

Considering the complexity of tumor immunity, the classification of tumor immune microenvironment based on the interaction between PD-1/PD-L1 and TILs will further deepen our understanding of the underlying mechanism and assist in identifying the best immunotherapy strategy for various tumor types. Preliminary studies suggest that tumors can be divided into four categories according to the presence of TILs and the PD-L1 expression levels: T1 (PD-L1^-^, TIL^-^), T2 (PD-L1^+^, TIL^+^), T3 (PD-L1^-^, TIL^+^), and T4 (PD-L1^+^, TIL^-^) (37). Importantly, this classification method will guide the selection of the appropriate treatment method for achieving the best therapeutic response for each tumor type. Below, we will briefly describe the appropriate immunotherapy strategy for different tumor types according to this classification method.

## Therapeutic Strategies for Different Tumor Types in HIS Mice

**Figure 2:**
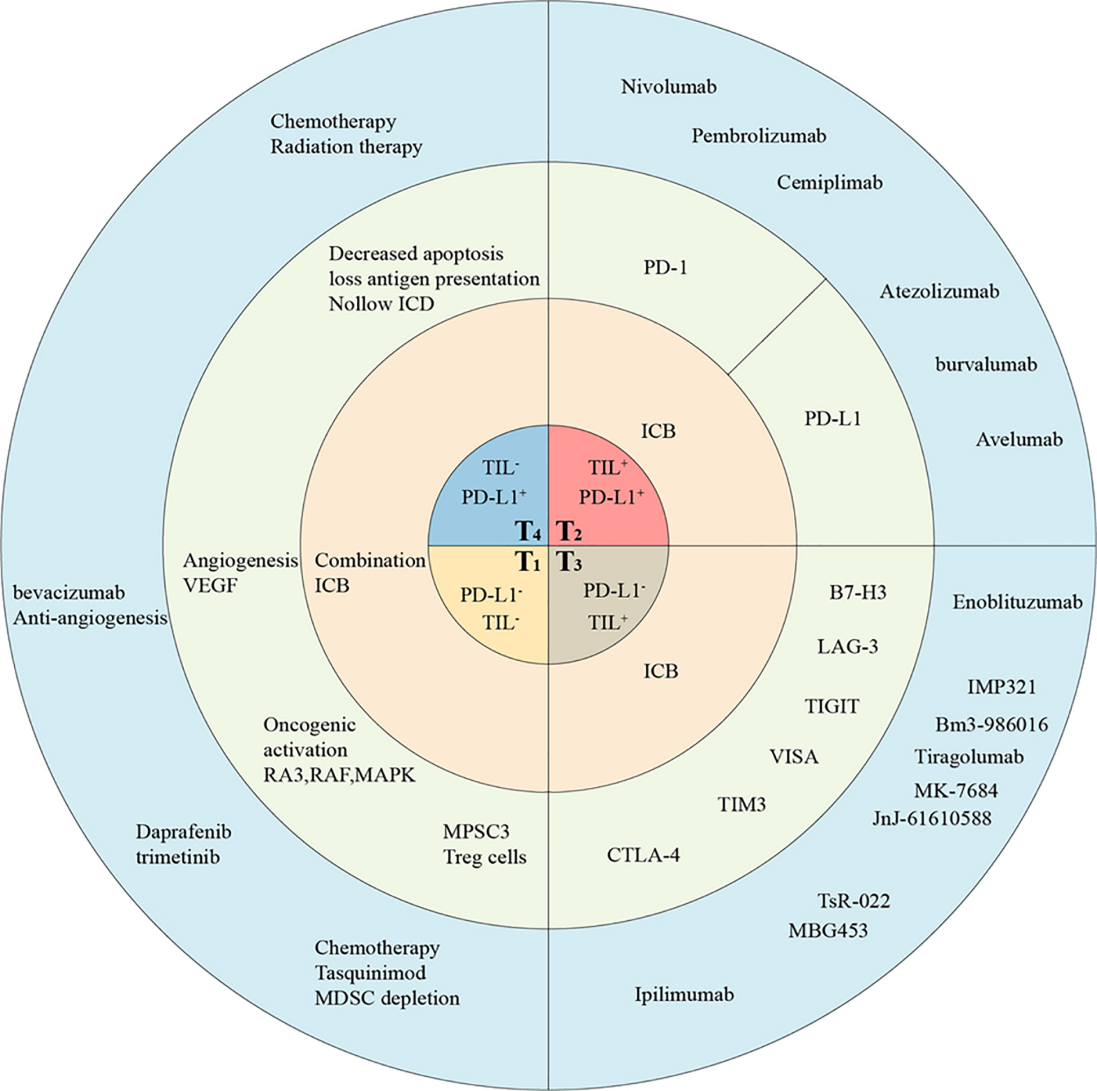
The classification of TMEs in humanized mice allow tailored cancer immunotherapeutic strategies. Tumors are classified into four different types based on the presence of TILs and their PD-L1 expression, including T1 (TIL^-^ PD-L1^-^), T2 (TIL^+^ PD-L1^+^), T3 (TIL^+^ PD-L1^-^), and T4 (TIL^-^ PD-L1^+^). This proposed framework of stratifying tumors in humanized mice provides a reference for determining the strategies best suited for targeting the four different tumor classes. The main treatment strategies (in pink), potential targets (in gray), and drugs (in blue) have been listed. ICB, immune checkpoint blockade; ICD, Immunogenic cell death.

T2 tumors, characterized by a large number of infiltrated immune cells and high PD-L1 expression level, responded the best to ICI treatment. Although lacking PD-L1 expression, T3 tumors might be immunosuppressed *via* other ICI. In T1 and T4 tumors, because of the lack of immune cell infiltration, the effect of anti-PD-1/PD-L1 therapy is poor; however, the tumors can be treated by combining the ICI treatment with other methods such as chemotherapy, radiotherapy, and targeted therapy. These combined approaches would increase immune infiltration in the tumor, thus further utilizing anti-PD-1 or PD-L1 antibody treatment (**Figure 2**).

### Treatment for T2 and T3 Tumors

ICI therapy displays the most effective response in T2 (PD-L1^+^, TIL^+^) melanoma and NSCLC tumors because of their large number of infiltrated immune cells and high PD-L1 expression levels. Approximately, 38% of patients with advanced melanoma exhibit T2 tumors and benefit from anti-PD-1/L1 monotherapy (38). In fact, a large number of anti-PD-L1 antibodies have been used in humanized mice. Using an Hu-CD34^+^ HSC mouse model loaded with lung cancer PDX, researchers discovered high immune cell infiltration in the tumor. Further treatment with pembrolizumab or nivolumab significantly inhibited the growth of tumors and was accompanied by increased cytotoxic T lymphocytes and the decrease of myeloid-derived suppressor cells (MDSCs) (25). In another experiment, Lin et al. detected a large number of T cells infiltrated within the tumor as well as high PD-L1 expression levels in an Hu-PBMC mouse model loaded with NSCLC cells, and further anti-PD-1 treatment displayed positive effects (10). To further clarify the relationship between immune cell infiltration within tumors and the effect of immunotherapy, the researchers transplanted cell lines such as of lung cancer, hepatocellular carcinoma, adrenocortical cancer, breast cancer, as well as colon cancer into Hu-CD34^+^ HSC mice and treated them with ICI (23, 25, 39, 40). The results showed that the treatment was effective only in tumors exhibiting high CD45^+^ T cell infiltration (24). This suggested that humanized mice can be used for tumor classification and direct immunotherapy evaluation of T2 tumors. Thus, it is important to correctly identify the tumor type and treat patients exhibiting this type of tumors to avoid the additional toxicity and the cost of combined immunotherapy.

Partial T3 tumors display a poor response to ICI therapy targeting PD-1/PD-L1, suggesting that this type of tumor may be immunosuppressed *via* other ICI therapies. Ipilimumab, a monoclonal antibody targeting CTLA-4, has achieved good therapeutic effects in T3 melanoma (41). Furthermore, Ipilimumab has also been used for the treatment of humanized mice exhibiting T3 tumors and showed good therapeutic effects (42). Lastly, some new immune checkpoint targets have been identified in T cells, including LAG-3, TIM-3, TIGIT, VIST, and B7-H3. All of these emerging candidate immune targets are either being assessed in clinical trials or under active development.

#### LAG-3

As early as 1990, Triebel et al. discovered LAG-3 (CD223), a molecule usually expressed in activated CD4^+^ and CD8^+^ T cells, regulatory T cells (Tregs), NK cell subsets, B cells, and dendritic cells (DCs). LAG-3 inhibits the activation, proliferation, and cytokine secretion of helper T cell 1 (Th1) (43). In view of its immunosuppressive effect, at least 10 types of LAG-3 blockers have been developed and studied in clinical trials; however, the results have not yet been reported. Therefore, it is of great significance to study the targeting of LAG-3 for treating tumors exhibiting low PD-L1 expression levels in an HIS mouse model. In addition, numerous preclinical mouse models have shown that PD-1/PD-L1 blockade can upregulate LAG-3 or other immune checkpoint molecules as a compensation mechanism (44). These findings indicate that these two types of ICI treatments can be combined in preclinical models and clinical trials. In fact, bispecific monoclonal antibodies against LAG-3/PD-(L)1, such as FS118, MGD013, and TSR-003, have already been developed (45). In a mouse model exhibiting an HIS and being inoculated with NSCLC, TSR-033 enhanced the efficacy of PD-1 monotherapy, while increasing the immune T-cell activation and proliferation and eliciting durable immunologic memory upon tumor rechallenge (42).

#### TIM-3

TIM-3 is also known as hepatitis A virus cell receptor 2 (HAVCR2). As early as 2002, TIM-3 was identified as a protein selectively expressed in CD4^+^ Th1 and CD8^+^ cytotoxic T cells (46). However, TIM-3 is currently classified as an immune checkpoint molecule similar to CTLA-4 or PD-1. Numerous experiments have shown that high TIM-3 expression levels are associated with poor solid malignant tumor prognosis (47). Preclinical experiments have confirmed that blocking TIM-3 alone or in combination with PD-1 blocking can limit tumor growth by regulating the TME (48, 49). Clinical trials of therapies targeting TIM-3 and both TIM-3 and PD-1 are currently underway. As in the case of LAG-3, a bispecific antibody targeting both PD-1 and TIM-3 named RO7121661 was developed by Roche; this antibody is currently under evaluation in a phase I clinical trial for treating advanced solid tumors (NCT03708328) (50). Therefore, the treatment of tumors exhibiting low PD-L1 expression levels using an anti-TIM-3 monoclonal antibody alone or in combination with an anti-PD-(L)1 monoclonal antibody could be evaluated in HIS mouse models.

#### TIGIT

TIGIT, a T cell immunoglobulin and ITIM domain, is an immune checkpoint target first discovered by Yu et al. in 2009 (51). Initial studies suggested that TIGIT indirectly inhibits T cell activation; however, subsequent studies have shown that TIGIT can also directly inhibit T cell function by competing with CD226 (52). It has recently been reported that TIGIT simultaneously blocks other checkpoint receptors, such as PD-1 and TIM-3 and has synergistic regulatory and antitumor effects (53). Numerous preclinical trials have achieved some positive results using TIGIT therapy, while the clinical effects of single drug treatment and a combined therapy with anti-PD-1 monoclonal antibodies in patients with advanced malignant tumors are under investigation. Other drugs, such as BMS-986207 (Bristol-Myers Squibb, New York, NY, USA) and AB-154 (Arcus Biosciences, Heyward, CA, USA), have also entered phase I clinical trials; however, no clinical results have been reported so far (54). Nevertheless, blocking these emerging immune checkpoint targets to treat T3 tumors in HIS mice models is extremely promising. In addition, apart from monotherapy, further attempts have been made to design a reasonable combination of immunotherapy to achieve synergistic inhibition of tumor growth.

### Treatment of T1 and T4 Tumors

In tumors lacking immune cell infiltration (T1 and T4), the effect of anti-PD-1/PD-L1 therapy is poor owing to the absence of an antitumor immune response. The lack of TILs in these tumors may be caused by a variety of factors, ranging from impaired tumor antigen presentation to the inhibition of immune cell activation or transport. Thus, most solid tumors are classified as T4 (PD-L1^+^, TIL^-^). However, the PD-L1 expression level alone cannot be used as a predictor of anti-PD-1 or anti-PD-L1 response in T4 tumors because blocking PD-1 or PD-L1 in these tumors is unlikely to induce a T cell response in the absence of infiltrated TILs. A good strategy is to increase immune infiltration in the tumor through a combination of treatments, followed by treatment with anti-PD-1 or PD-L1 antibodies. For instance, previous studies showed that Hu-CD34^+^ HSC mice bearing melanoma cell line A2058 were non-responsive to pembrolizumab monotherapy owing to the lack of immune cell infiltration. Furthermore, they showed that ONCOS-102 (an oncolytic adenovirus armed with Human GM-CSF and an Ad5/3 chimeric capsid) treatment promoted the infiltration of CD8^+^ T cells in the tumor, and its combined treatment with pembrolizumab significantly inhibited tumor growth (55). Clinically, traditional methods such as chemotherapy, radiotherapy, and targeted therapy are reportedly effective for promoting the activation of the above pathways, and thus may provide a theoretical basis for combined therapy in an HIS mouse model.

#### Immunotherapy Combined With Chemotherapy

Chemotherapy-induced immune activation is an effective supplemental strategy to immunotherapy. In addition to directly killing tumor cells, chemotherapy can enhance the antitumor immune response by changing the immunogenicity of tumor cells, thus regulating immune cell subsets and the TME. The damage-associated molecular patterns produced by tumor cells killed by chemotherapeutic drugs can activate the T cell-mediated adaptive antitumor immune response in a process known as immunogenic cell death (56). Moreover, chemotherapy increases number of tumor killer immune cells of various subsets. Using a mouse bearing breast cancer, Alizadeh et al. demonstrated that adriamycin treatment selectively reduced MDSC number in the spleen, peripheral blood, and tumors, while increasing the ratio of CD4^+^ and CD8^+^ T lymphocytes/MDSCs (57). Using an Hu-CD34^+^ HSC mouse model bearing the triple-negative breast cancer cell line MDA-MB-231, researchers have shown that cyclophosphamide can increase the ratio of CD8^+^/Treg in TILs, thereby promoting subsequent immunotherapy effects (58). In clinical practice, platinum neoadjuvant chemotherapy was administered to patients with advanced cervical cancer, and immune infiltrating cells were observed inside and around the tumor. It was found that the number of Treg cells in and around the tumor significantly decreased, whereas the number of CD8^+^ T cells remained unchanged. Furthermore, multivariate analysis demonstrated that the increase of CD8/peritumor Foxp3 expression levels in the intratumoral region indicated good clinical outcomes (59). In summary, these observations support the hypothesis that chemotherapy not only inhibits tumors but also participates in the active regulation of the immune system. Clinically, the successful application of anti-PD-1 therapy combined with chemotherapy for metastatic NSCLC also demonstrated the potential of this dual therapy (60). Therefore, investigating the effects of specific chemotherapeutic drugs alone and in combination with immunotherapy in HIS mouse models could significantly guide future clinical trials.

#### Immunotherapy Combined With Radiotherapy

It has been reported that radiation not only eliminates local tumors but also leads to the regression of untreated distal metastases. For the first time, this process has been described as an abscopal effect, and there is increasing evidence that this abscopal effect is likely to be immune-mediated, mainly in a T cell-dependent manner (61). Preclinical studies showed that the efficacy of combined radiation therapy (RT) and ICI treatment is related to the immune cell regulation in the TME. The combined therapy increased the number of IFN-γ^+^-TNF-α^+^-CD8^+^ T cells and the expression levels of PD-1, TIM-3, and LAG-3 in CD8^+^ T cells (62). The maintenance of increased co-expression levels of PD-1 and TIM-3 in CD4^+^ T cells, CD8^+^ T cells, and Tregs ultimately leads to T cell exhaustion. Following ICI addition to the RT, the antitumor response was enhanced by restoring exhausted CD8^+^ TIL activity and increasing the CD8^+^ T cell/Treg ratio in several mouse tumor models as compared to those of the control groups (63). However, as all ICI used in these preclinical models targeted mouse immune checkpoints, combined therapies in HIS mice models will provide more convincing evidence of these effects.

#### Immunotherapy Combined With Targeted Drug Therapy

Recently, the recognition of uncontrolled cellular signal pathways that led to tumorigenesis has promoted the successful development of molecular targeted therapy. DNA repair and angiogenesis pathways have provided effective treatment options for patients with different types of malignant tumors. Several studies have shown that these pathways also display immunomodulatory effects on systemic and intratumoral antitumor immune responses; thus, suggesting that the combination of molecular targeted therapy and ICI treatment might elicit synergistic antitumor effects and can be explored in HIS mice for producing synergistic antitumor effects (64).

#### Immunotherapy Combined With Carcinogenic Pathway Inhibitors

Abnormal regulation of the RAS/RAF/MAPKA signal pathways is common in tumor transformation and tumorigenesis. Mutations of the *BRAF* gene are associated with a variety of cancers, especially melanoma (65). In the melanoma cell model, the BRAFV600 mutation leads to a decline in antitumor immune function by increasing the expression levels of immunosuppressive factors such as interleukin (IL)-10 and vascular endothelial growth factor (VEGF) and upregulating PD-L1 expression (66). The combination of BRAF inhibitors and anti-CTLA-4 antibodies has been investigated in multiple clinical trials. In a phase 1 clinical trial, combined application of daprafenib, trametinib, and duvacizumab displayed an apparent therapeutic effect (67). In an ongoing clinical study, researchers utilized pebrizumab combined with daprafenib and trametinib in the attempt of treating metastatic melanoma. A high T cell infiltration rate was observed in tumor biopsy within one week of administering the BRAF/MEK inhibitor; however, the T cell infiltration rate decreased after 2 weeks of treatment. These findings indicated that the timing of combination treatment with anti-PD-1 may affect patient prognosis (68). Therefore, using the HIS model, we can explore the effects of BRAF/MEK inhibitors on the immune system, using different concentrations and treatment durations, thus allowing the identification of the best combined treatment strategy and providing theoretical evidence for future clinical trials.

#### Immunotherapy Combined With DNA Repair Inhibitors

DNA damage repair plays an important role in cell cycle regulation and tumorigenesis. Inhibition of this process might increase the mutation burden of tumors, and a high mutation load has been shown to be associated with the clinical benefits of immune checkpoint blocking therapy for lung cancer and melanoma (69). Therefore, theoretically, increasing the new antigen load in tumor cells can be used in combination with subsequent checkpoint inhibition, especially in tumors with high endogenous DNA damage (70). Poly ADP-ribose polymerase (PARP) has a large effect on the repair of single strand DNA breaks. In preclinical trials, PARP inhibitors have been shown to slow down chronic inflammation and promote T cell infiltration (71). Several clinical trials investigating the combination of different PARP inhibitors with ICI are currently being conducted in a variety of solid tumors, including NSCLC and breast cancer. Nevertheless, HIS mice models can be used to better understand the interaction between DNA damage repair and immunosuppression.

#### Immunotherapy Combined With Targeted VEGF Therapy

Increasing evidence suggests that antiangiogenic agents stimulate the immune system, and that immunotherapy might also exhibit antiangiogenic effects. It has been shown that VEGF weakens the antitumor immune response through two main modes of action: first, VEGF was shown to block the infiltration of T cells into the tumor by inhibiting the adhesion of lymphocytes to activated endothelial cells; and second, VEGF was shown to promote the function of immunomodulatory cells through a variety of mechanisms (72). Considering the immunosuppressive and angiogenic effects of VEGF in tumors, it is not surprising that antiangiogenic agents stimulate the immune response and enhance the effectiveness of immunotherapy. In a colon adenocarcinoma model, the simultaneous inhibition of PD-1 and VEGFR2 using monoclonal antibodies significantly inhibited tumor growth without exerting increased toxicity as compared to that of monotherapy (73). Another study reported that the triple therapy of C118-9, bevacizumab, and pembrolizumab inhibited tumor growth in a humanized mouse model loaded with hepatocellular carcinoma cell lines or PDX. Furthermore, they showed that the observed effects were explained by the single-drug or dual-drug combination therapy strategies (74). This evidence led to clinical studies exploring the possibility of a combination of antiangiogenic treatments and immunotherapy for treating NSCLC.

Additionally, some new immunotherapies, such as adoptive cell therapy (75), oncolytic virus therapy (76), and tumor vaccines (77) can be used to change the TIME. Their effects were examined in combination with IC inhibitors in HIS mouse models.

## Discussion

In summary, tumor immunotherapy is one of the most promising directions in the field of tumor therapy, while the HIS mouse model provides sufficient preclinical evidence for the benefits of immunotherapy. Nevertheless, diversified immunotherapy schemes pose a challenge to the development of humanized mice, as the process of creating an improved simulation of the HIS became essential for further drug development. Most importantly, the tumors were classified in four basic classes in relation to the type and degree of immune cell infiltration within the TME in HIS mouse bearing humanized tumor, and this classification can be used to select the combination of chemotherapy and immunotherapy drugs that might lead to the most efficient tumor treatment. Here, we proposed different combination treatment strategies to enhance the effect of immunotherapy and provides effective experimental schemes for tumor immunotherapy testing in HIS mouse models.

Although these recent studies are interesting and promising, numerous ambiguities and deficiencies associated with immunotherapy remained unaddressed. i) The underlying mechanisms of ICI treatment resistance are not yet fully understood. We need to clarify how is the human immune system affected by different TMEs and how to proceed to overcome the resistance of immune checkpoint blockade and guide reasonable combined PD-1 therapy. ii) The best combination therapy strategies to improve efficacy are unclear. While some patients develop autoimmune reactions to the therapy, there are few strategies to improve antitumor effects while reducing immune-related adverse events. iii) The appropriate time point to block PD-1/PD-L1, especially for combination therapy should be confirmed. By identifying the best treatment time window, the response rate might be improved, the drug dose reduced, and ultimately, the therapy safety consolidated. iv) A more representative preclinical model is needed to promote the translation of experimental results into clinical practice. There are no precise predictive biomarkers that allow the distinction between responders and non-responders to the combination therapy, while the expression levels of PD-L1 and TILs cannot completely predict the responses of patients to immunotherapy. Therefore, it is important to use HIS mice models to screen precise biomarkers of immunotherapy efficacy. In view of these individual differences, precision medicine is required to construct an HIS mouse model loaded with both PDX and immune cells from the same patient, thus allowing the evaluation of the personalized effects of immunotherapy. Taken together, considering the rapid development of immunotherapy, it is of great importance to select a suitable HIS mouse model for clinical transformational research and provide a theoretical basis for clinical decision-making.

## Author Contributions

WG, JZ, and CS: conceptualization. WG, CZ, TQ, and CS: writing. WG, and CS: revising. All authors contributed to the article and approved the submitted version.

## Funding

This study was funded by the National Natural Science Foundation Program of China (No. 31772546 and 32070532) and Shaanxi Natural Science Foundation Program No. 2020PT-005.

## Conflict of Interest

The authors declare that the research was conducted in the absence of any commercial or financial relationships that could be construed as a potential conflict of interest.

## Abbreviations

HIS, human immune system; ICI, immune checkpoint inhibitor; PBMC, peripheral blood mononuclear cells; HSC, hematopoietic stem cell; PDX, patient-derived xenograft; TME, tumor microenvironment; MDSCs, myeloid-derived suppressor cells; VEGF, vascular endothelial growth factor; TILs, tumor-infiltrating lymphocytes.
